# Lipopolysaccharide affects metabolic processes and energy homeostasis in the corpus luteum

**DOI:** 10.3389/fmolb.2024.1523098

**Published:** 2025-01-07

**Authors:** Karol Mierzejewski, Robert Stryiński, Iwona Bogacka, Monika Golubska, Mónica Carrera, Aleksandra Kurzynska

**Affiliations:** ^1^ Department of Animal Anatomy and Physiology, Faculty of Biology and Biotechnology, University of Warmia and Mazury in Olsztyn, Olsztyn, Poland; ^2^ Department of Biochemistry, Faculty of Biology and Biotechnology, University of Warmia and Mazury in Olsztyn, Olsztyn, Poland; ^3^ Department of Food Technology, Institute of Marine Research (IIM), Spanish National Research Council (CSIC), Vigo, Spain

**Keywords:** inflammation, mid-luteal phase, pig, glucose metabolism, progesterone, steroidogenesis

## Abstract

**Introduction:**

Chronic inflammation caused by *Escherichia coli* infections has a significant negative impact on the reproductive system and impairs fertility. The corpus luteum (CL) plays a central role not only in regulating the ovary cycle, but also in implantation of the embryo and maintenance of early pregnancy through the secretion of progesterone. Understanding the intricate interplay between inflammatory processes and reproductive organ’s function is crucial for the development of effective therapeutic strategies to alleviate reproductive disorders and improve fertility.

**Methods:**

The aim of this study was to determine the in vitro effects of lipopolysaccharide (LPS) on the proteomic profile of the porcine CL in the mid-luteal phase of the estrous cycle using LC-MS/MS analysis. The CL slices were incubated in the presence of LPS for 24 h.

**Results:**

We identified 12 differentially regulated proteins after treatment with LPS (7 of them were upregulated, while 5 were downregulated). The analysis showed that these proteins are involved in processes such as glucose metabolism, the tricarboxylic acid cycle (TCA), detoxification processes as well as steroid biosynthesis in the CL. Moreover, we demonstrated that LPS decreases glucose levels and increases progesterone levels in the CL.

**Conclusion:**

These findings suggest that LPS modulates key metabolic pathways in the CL, potentially impacting its functional activity.

## 1 Introduction

The corpus luteum (CL) is a heterogeneous endocrine gland that arises cyclically from the follicular cells of the ovaries in mature females during the luteinization process. The theca cells of the follicle luteinize into small luteal cells and the granulosa cells into large luteal cells. During pregnancy, the CL remains in the ovary until birth, while it regresses if fertilization does not occur (Stocco et al., 2007). Maintaining the activity of the CL during early pregnancy is the most important function of the reproductive system (Wiltbank et al., 2018). The main function of the CL is to synthesise various hormones, including progesterone (P4) and prostaglandins (PGs) – luteotropic PGE2 and luteolytic PGF2α, which regulate tissue activity via autocrine and paracrine mechanisms and are essential for the proper course of the estrous cycle and the successful development of pregnancy (Wehling and Lösel, 2006; Zerani et al., 2021).

One of the factors that lead to reproductive system disorders is inflammation, which is a natural and necessary response to pathological situations such as bacterial infections. Nevertheless, if this inflammatory process continues unchecked, it can develop into chronic inflammation leading to organ dysfunction (Akira et al., 2006). The occurrence of a chronic inflammatory response in the female reproductive system frequently correlates with the presence of lipopolysaccharide (LPS), an endotoxin produced by Gram-negative bacteria such as *E. coli* (*Escherichia coli*) (Jana et al., 2007). The inflammatory process contributes to a significant percentage of gynaecological diseases, especially in females and animals of reproductive age, and impairs reproductive processes (Weiss et al., 2009). It has been reported that chronic inflammation can lead to anovulation, infertility, miscarriages, and pregnancy complications (Boots and Jungheim, 2015). Although there is evidence that inflammation interferes with reproductive processes, there is still lack of information on the effects of inflammation on the CL function. It should be emphasized that the similarities between the reproductive systems of pigs and humans, in particular the physiological and anatomical features as well as hormone regulation, steroidogenesis and luteal function, make the pig a good model for the study of reproductive processes (Mordhorst and Prather, 2017; Lunney et al., 2021). Furthermore, the systemic immune responses of pigs are comparable to those of humans, making them a valuable model for studying the effects of various stimuli such as LPS on reproductive health (Meurens et al., 2012; Lunney et al., 2021). In addition, pigs show a high degree of sequence and chromosome structure homology with humans, and numerous studies have highlighted their importance for proteomics research (Verma et al., 2011; Bassols et al., 2014). Our previous study shows the modulating effect of LPS on the expression of genes that control the immune response in pigs, as well as the activity of oxidoreductases and antioxidant enzymes (Mierzejewski et al., 2023b). This study was undertaken to determine the *in vitro* effect of LPS on the global proteomic profile of the porcine CL during the mid-luteal phase of the estrous cycle using high resolution LC-MS/MS analysis.

## 2 Materials and methods

### 2.1 Experimental animals

**FIGURE 1:**
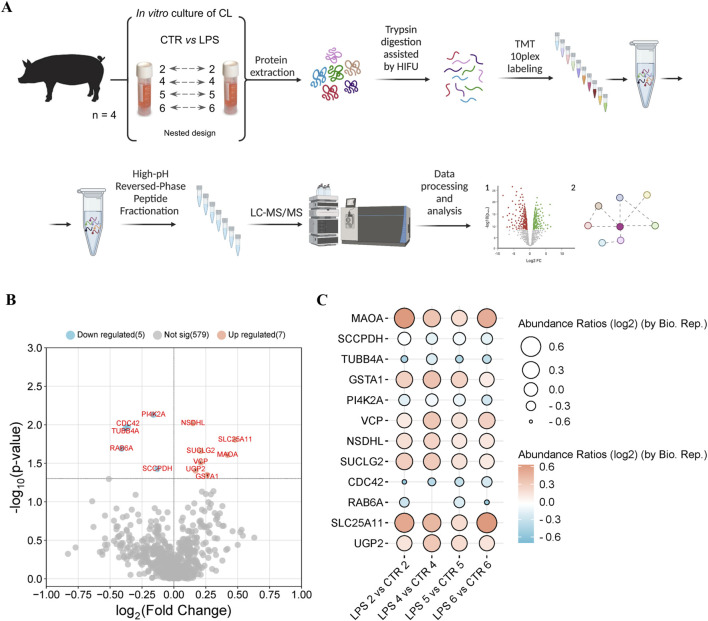
**(A)** Experimental setup and workflow for the comparison of the proteomes of the corpus luteum of pigs with and without LPS treatment. A total of 4 pigs were used and the corpus luteum of the same pig was used as control and after inflammation induction with LPS as treated tissue (nested design). Samples were collected, proteins extracted and subjected to trypsin digestion stimulated with high-intensity focused ultrasound (HIFU). The peptides from each sample were labelled with tandem mass tags (TMTs). After labelling, samples were pooled and fractionated using the high pH reverse phase fractionation method. Tandem mass spectrometry data were acquired using an LTQ-Orbitrap Elite spectrometer and proteins were identified using Proteome Discoverer 2.4. The identified proteins were subjected to statistical analysis and the results obtained were visualized. **(B)** The volcano plot of differentially regulated proteins (DRPs) between the proteomes of the corpus luteum of pigs with and without LPS treatment (FC ≥ 1, p-value ≤ 0.05). **(C)** The balloon plot of DRPs identified between the corpus luteum of pigs with and without LPS treatment. Balloon size and colors were used to indicate the abundance ratio values (log2) for each biological replicate. The detailed list of DRPs can be found in Supplemental Table 2.

The experiment was carried out on corpora lutea obtained from female pigs designated for commercial slaughter and meat processing in accordance with national guidelines for animal care (Act of 15 January 2015 on the protection of animals used for scientific or educational purposes and Directive 2010/63/EU of the European Parliament and of the Council of 22 September 2010 on the protection of animals used for scientific purposes). The CL were obtained from mature female crossbred pigs (Large White and Polish Landrace), at 7 months of age with a body weight of approximately 100 kg (n = 4) during days 10–12 of the estrous cycle, which correspond to the mid-luteal phase of the estrous cycle (Figure 1A). The pigs were observed over two consecutive heat cycles on the farm. The onset of heat, characterized by the behaviour of the sows in the presence of a boar, was defined as day 0 of the estrous cycle. Following observation, the animals were transported to a local slaughterhouse, where the ovaries were immediately dissected. The excised tissue was then transferred to the laboratory on ice and immersed in phosphate-buffered saline (PBS) with an antibiotic cocktail (100 IU/mL penicillin and 100 mg/mL streptomycin, sourced from PolfaTarchomin, Poland). In the laboratory, the phase of the estrous cycle was confirmed based on the morphological characteristics of the ovaries (Mierzejewski et al., 2023b).

### 2.2 *In vitro* incubation

In the laboratory, the CL were dissected from the ovary, connective tissue was removed and placed on ice in a sterile Petri dish. The CL were cut into small pieces (100 ± 10 mg, in duplicate from each experimental replicate) as previously described (Kunicka et al., 2024; Mierzejewski et al., 2023a). Each tissue explant was placed in M199 medium (Sigma Aldrich, St. Louis, MO, United States) supplemented with 0.1% BSA fraction V (Roth, Germany) and antibiotics. The explants were pre-incubated for 2 h in a water bath at 37°C in an atmosphere of 95% O_2_ and 5% CO_2_, and then treated with LPS (100 ng/mL, from *E. coli*) for 24 h. The dose of LPS was selected based on literature data and our previous study (Mierzejewski et al., 2023b). Explants not treated with LPS were considered as controls. After the incubation, tissue explants were frozen at −80 °C until further analysis.

### 2.3 Protein isolation

The protein extracts were obtained from the CL explants that were previously incubated *in vitro*. The tissue was mechanically homogenised (TissueRuptor II, Qiagen, Hilden, Germany). Protein extraction was performed with lysis buffer (60 mM Tris–HCl pH 7.5, 1% lauryl-maltoside, 5 mM PMFS and 1% DTT) as previously described (Kunicka et al., 2024). Protein concentration was quantified using the bicinchoninic acid method (Pierce BCA Protein Assay Kit, Thermo Fisher Scientific, Waltham, MA, United States) according to the manufacturer’s protocol. SDS-PAGE electrophoresis (in a polyacrylamide gel containing 12% acrylamide) was performed as a control step as previously described (Mierzejewski et al., 2021). Gels were stained with silver using the Pierce Silver Stain for Mass Spectrometry Kit (Thermo Fisher Scientific, Waltham, MA, United States). Then, a total of 100 μg of protein from each sample was transferred to a new tube and methanol/chloroform precipitation was performed. Subsequently, ultrafast tryptic digestion was carried out with simultaneous application of high-intensity focused ultrasound (HIFU) as previously described (Stryiński et al., 2019).

### 2.4 TMT labelling and pH reversed phase fractionation

Labelling and fractionation procedures were performed as previously described (Mierzejewski et al., 2021). In brief, TMT 10-plex isobaric label reagents (0.8 mg, Thermo Fisher Scientific) were resuspended in 41 μL of anhydrous acetonitrile and added to 100 μg of protein digest according to the kit manufacturer’s instructions. Samples were labelled with TMT 10-plex (nested design; CTR: pig no. 2–126, pig no. 4–127C, pig no. 5–128C, pig no. 6–129C; LPS: pig no. 2–127N, pig no. 4–128N, pig no. 5–129N, pig no. 6–130N) and combined in a new tube in equal amounts and the reaction was carried out for 1 h at room temperature according to the manufacturer’s instructions. TMT-labelled peptide concentration in combined sample was measured using a Pierce Quantitative Colorimetric Peptide Assay (Thermo Fisher Scientific) according to the kit manufacturer’s instructions. To increase the number of peptide identifications, the combined sample was fractionated with a Pierce High-pH Reversed-Phase Peptide Fractionation Kit (Thermo Fisher Scientific, Waltham, MA, United States) according to the manufacturer’s instructions. In brief, a total of 100 μg of digested peptide was dissolved in 300 μL of 0.1% TFA solution. Eight different fractions were obtained stepwise using the appropriate elution solutions according to the kit manufacturer’s instructions. The peptide content of each fraction was determined calorimetrically using the Quantitative Colorimetric Peptide Assay (Thermo Fisher Scientific), and stocks of 1 μg were evaporated to dryness by vacuum centrifugation (SpeedVac concentrator, Thermo Fisher Scientific). Samples were stored at −80°C until further analysis.

### 2.5 LC-MS/MS analysis

Eight peptide samples (1 μg each), obtained after fractionation, were acidified with 0.1% formic acid, and analysed by LC-MS/MS using the Proxeon EASY-nLC II liquid chromatography system (Thermo Fisher Scientific) coupled to an LTQ-Orbitrap Elite mass spectrometer (Thermo Fisher Scientific). Peptide separation was performed as described by Stryiński et al. (2019), Stryiński et al. (2022). All acquired MS/MS spectra were analysed using SEQUEST-HT (Proteome Discoverer 2.4 package; Thermo Fisher Scientific, Waltham, MA, United States) against a reference proteome of *Sus scrofa* available in the UniProt/TrEMBL database (downloaded in January 2024; proteome ID: UP000008227; # of entries 46,176). The following restrictions were used: full tryptic cleavage with up to 2 missed cleavage sites and tolerances of 10 ppm for parent ions and 0.06 Da for MS/MS fragment ions. TMT labelling (+229.163 Da on N-termini and lysine residues) and carbamidomethylation of cysteine (+57.021 Da) were set as fixed modifications. The permissible variable modifications were methionine oxidation (+15.994 Da), acetylation (+42.011 Da) of the N terminus of the protein, and deamidation (+0.984 Da) of asparagine and glutamine. Moreover, searching parameters included four maximal dynamic modification sites.

### 2.6 Statistical analysis

The results were subjected to statistical analysis to determine the peptide false discovery rate (FDR) using a decoy database and the Target Decoy PSM Validator algorithm (Käll et al., 2007). The FDR was kept below 1%, and only proteins classified as master proteins were used for further analysis. Relative quantification (RQ) was performed using the Quantification Mode and normalization against total peptide amount (Proteome Discoverer 2.4 package, Thermo Fisher Scientific, Waltham, MA, United States). After RQ, several filters were applied to obtain the final list of differentially regulated proteins (DRPs): (a) ≥ 1.1-fold change in normalized ratios (LPS 2, 4, 5, 6 vs CTR 2, 4, 5, 6, respectively), and (b) nested ANOVA and Tukey HSD *post hoc* test (p-value ≤ 0.05).

### 2.7 Biochemical analyses

The experimental material for glucose and progesterone measurements was obtained from the same CL used for proteomic studies. The CL were weighed and placed in PBS at a ratio of 1:10 (weight/volume). The tissue was thoroughly homogenised until a uniform suspension was achieved. After homogenisation, the samples were centrifuged at 5,000 g for 10 min at 4°C to remove debris and insoluble material. The supernatant was collected for further analysis. Glucose content was determined by the Trinder method (Trinder, 1969) using the Glucose Oxidase Reagent Set (Pointe Scientific, Poland) according to the manufacturer’s instructions. Three technical replicates of each biological replicate were performed. Absorbance values were measured at 500 nm using the UV-3100PC spectrophotometer (VWR International, Poland) in comparison to the reagent blank. Glucose levels were calculated using the standard curve generated for serial dilution of the glucose standard (100 mg/dL). The concentration of progesterone was determined using a commercially available ELISA kit (Reed Biotech, cat: RE10118) according to the manufacturer’s protocol. The range of the standard curves was 78.13–5,000 pg/mL. Absorbance values were measured at 450 nm using the Infinite M200 Pro Reader with the Tecan i-control software (Tecan, Switzerland). Statistical analyses of the results obtained were performed using the t-test in Prism 8 software (GraphPad Software, Inc.).

## 3 Results

### 3.1 Specific proteome changes in the porcine CL after LPS treatment

As a result of the LC-MS/MS analysis, we identified in a total 591 proteins in the porcine CL under the influence of LPS during the mid-luteal phase of the estrous cycle compared to control (Supplemental Table 1). The volcano plot representation of DRPs is shown in Figure 1B. The most upregulated proteins are located towards the right side (red), while the most downregulated proteins are located towards the left (blue). Proteomic analysis showed that 12 proteins were differentially regulated in the LPS-treated CL compared to the control, of which 7 proteins were upregulated (Table 1; mitochondrial 2-oxoglutarate/malate carrier protein, amine oxidase, glutathione S-transferase, succinate-CoA ligase subunit beta, transitional endoplasmic reticulum ATPase, UTP-glucose-1-phosphate uridylyltransferase, NAD(P) dependent steroid dehydrogenase-like), and 5 proteins were downregulated (saccharopine dehydrogenase-like oxidoreductase, phosphatidylinositol 4-kinase type 2, cell division control protein 42 homolog, tubulin beta chain, RAB6A, member RAS onco family). Abundance ratios (log2) calculated in the nested design for each of the biological replicates LPS vs CTR, n = 4) are visualized in Figure 1C (Supplemental Tables 1, 2).

**TABLE 1:** Differentially regulated proteins in porcine corpus luteum under the influence of LPS (*p*-value ≤ 0.05).

Accession	Protein name	Gene name	Fold change	*p*-value	Regulation
A0A5G2QTH6	Amine oxidase [flavin-containing] A	MAOA	1.342	0.02466410	Up
A0A5G2QKI4	Saccharopine dehydrogenase-like oxidoreductase	SCCPDH	0.914	0.03744026	Down
A0A8W4F8R1	Tubulin beta chain	TUBB4A	0.770	0.01110213	Down
A0A5K1VST3	Glutathione S-transferase	GSTA1	1.196	0.04489194	Up
F1S8X7	Phosphatidylinositol 4-kinase type 2	PI4K2A	0.898	0.00741558	Down
F1SIH8	Transitional endoplasmic reticulum ATPase	VCP	1.153	0.03110576	Up
A0A287ASS9	NAD(P) dependent steroid dehydrogenase-like	NSDHL	1.108	0.00954027	Up
P53590	Succinate--CoA ligase [GDP-forming] subunit beta, mitochondrial	SUCLG2	1.158	0.02187344	Up
Q007T2	Cell division control protein 42 homolog	CDC42	0.779	0.01067093	Down
F1SUT0	RAB6A, member RAS onco family	RAB6A	0.752	0.02041883	Down
F1RFX9	Mitochondrial 2-oxoglutarate/malate carrier protein	SLC25A11	1.395	0.01564601	Up
A0A481C881	UTP--glucose-1-phosphate uridylyltransferase	UGP2	1.121	0.03938631	Up

### 3.2 DRPs are associated with diverse biological processes and metabolic pathways

**FIGURE 2:**
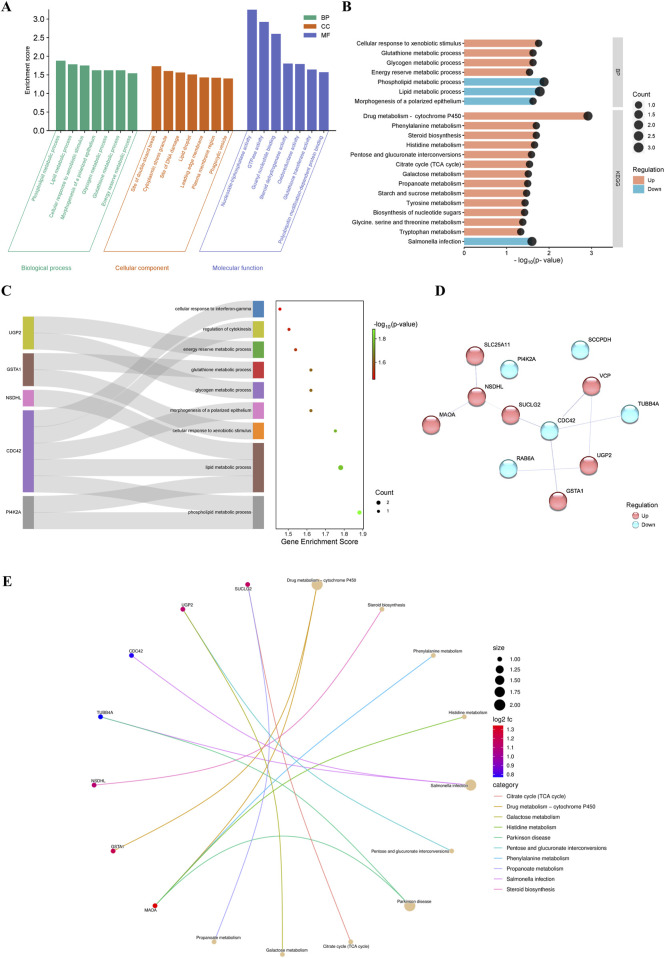
**(A)** Visualization of the results of the functional analysis. The results of the GO enrichment analysis. The 7 major GO subcategories assigned for the identified differentially regulated proteins (DRPs). **(B)** The results of KEGG metabolic pathways by identified DRPs. **(C)** The Sankey diagram represents proteins within selected biological processes. The right part is a dot plot; the dot sizes represent the number of proteins and the dot colors represent -log10 (p-values). **(D)** Protein–protein interaction network analysis of the DRPs between the proteomes of the corpus luteum of pigs with and without LPS treatment. The visualization was performed in Cytoscape v. 3.9.1. **(E)** Pathway cnetplot of the KEGG pathway enrichment analysis. DRPs are directly assigned to the pathways in which they are involved; the dot sizes represent the number of proteins and the dot colors the -log10 (p-values).

The 12 DRPs were assigned to functional ontology annotations. GO analysis divided the input proteins into three categories: MFs (19 different functions), BPs (88 different processes) and CC (17 different cellular components) (Supplemental Table 3). Selected seven top subcategories (p-value = 0.05) assigned for each of the three main GO categories are presented in Figure 2A. The functions assigned to the MF subcategory included nucleoside-triphosphatase activity (GO:0017111), GTPase activity (GO:0003924), and guanyl nucleotide binding activity (GO:0019001). The distribution of DRPs according to their abundance in cellular components was associated with cytoplasmic stress granule (GO:0010494), site of double-strand break (GO:0035861) and site of DNA damage (GO:0090734), as well as lipid droplet (GO:0005811), and phagocytic vesicle (GO:0045335). In the BP subcategory, most of the downregulated proteins were involved in lipid and phospholipid metabolic process (GO:0006629, GO:0006644), whereas the upregulated proteins were involved in the cellular response to xenobiotic stimulus (GO:0071466) and glycogen and glutathione metabolic processes (GO:0005977, GO:0006749) (Figures 2A, B). One of the most involved proteins was CDC42, which was assigned into 5 different biological processes (Figure 2C; Supplemental Table 3). CDC42 has been noted to have the most protein-protein interactions (4 interactions; Figure 2D). Based on the KEGG pathway analysis (Supplemental Table 4), where DRPs were assigned to 18 different metabolic pathways, most DRPs were involved in drug metabolism–cytochrome P450 (GSTA1, MAOA; ssc00982), steroid biosynthesis (NSDHL; ssc00100) and citrate cycle (SUCLG2; ssc00020) (Figure 2E). All these metabolic pathways were upregulated in response to LPS (Figures 2B, E).

### 3.3 LPS affects glucose and progesterone tissue level

**FIGURE 3:**
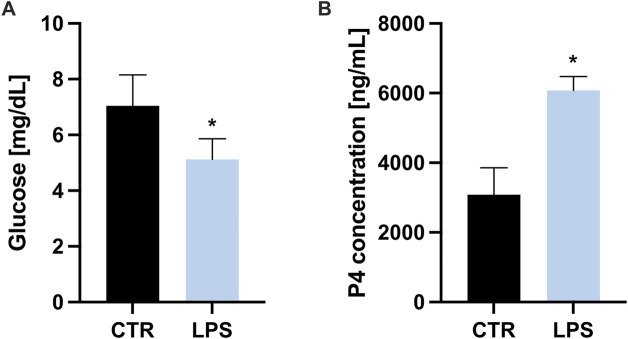
Comparison of **(A)** glucose and **(B)** progesterone (P4) concentration levels in control (CTR) and lipopolysaccharide-treated (LPS) groups in porcine corpus luteum during the mid-luteal phase of the estrous cycle.

Based on the results of the LC-MS/MS analysis, which demonstrated the modulatory role of LPS on the expression of proteins crucial for glucose metabolism and steroidogenesis, we further assessed the levels of glucose and progesterone in the CL tissue. The glucose content in the CL tissue was lower after LPS treatment–in the control group with an average of 5.124 mg/dL (SEM 0.73), in the LPS-treated group with an average of 7.05 mg/dL (SEM 1.1) (Figure 3A). In contrast, the concentration of progesterone in the homogenate of the CL tissue was significantly higher after LPS treatment – 3,085 ng/mL (SEM 1536.76) in the control group compared to 6,074.48 ng/mL (SEM 804.38) in the LPS-treated group (Figure 3B). The decrease in glucose levels may reflect a shift in metabolic activity, potentially due to increased local energy demand or altered glucose utilization. Conversely, the increase in progesterone concentration aligns with reports suggesting that inflammation can modulate steroidogenesis. These findings suggest a coordinated response of the CL to LPS, where metabolic changes may support or influence the altered hormonal activity.

## 4 Discussion

The proper functioning of the corpus luteum is crucial for successful reproduction. Since disruption of the luteal phase can affect fertility by preventing implantation and early development of the conceptus in livestock and humans, it is important to describe potential factors disrupting CL function. One of the factors that strongly interfere with reproductive functions is LPS. Here we showed that LPS altered the expression of 12 proteins in the CL during the mid-luteal phase of the estrous cycle; 7 of these proteins were upregulated, while 5 were downregulated. These include proteins involved in the citrate cycle (TCA cycle), glucose metabolism, steroid biosynthesis and detoxification processes.

The tricarboxylic acid cycle (TCA) is a major pathway for oxidative metabolism in the mitochondria. It is involved in macromolecule synthesis and redox balance maintenance and serves as a critical energy source for cells under aerobic conditions (Yang et al., 2014). There is increasing evidence that the metabolites of the TCA cycle significantly regulate immune responses. During cell stress, disrupted mitochondrial membranes release intermediates of the TCA cycle into the cytosol and thus influence cellular immunity. Our studies show a stimulatory effect of LPS on the expression of succinate-CoA ligase GDP-forming subunit beta (SUCLG2) in the porcine CL. This key enzyme of the TCA cycle is involved in the reversible conversion of succinyl-CoA to succinate and simultaneously catalyzes the phosphorylation of GDP to GTP at the substrate level (Van Hove et al., 2010). Succinate has been reported to be present at elevated levels during inflammation (Choi et al., 2021). In addition, succinate has been identified as a metabolite in innate immune signaling that increases interleukin-1β production during inflammation and plays a key role in macrophage activation in response to LPS stimulation (Tannahill et al., 2013; Mills and O’Neill, 2014). Our results contribute to the growing body of evidence elucidating the interplay between metabolic pathways and the inflammatory response. They suggest that the upregulation of SUCLG2 by LPS in the CL may indirectly mediate the inflammatory response.

Interestingly, our study also revealed a stimulatory effect of LPS on the expression of NAD(P)-dependent steroid dehydrogenase-like (NSDHL) – a sterol decarboxylase involved in cholesterol biosynthesis by catalysing the conversion of 4-methylzymosterol-carboxylate to 3-keto-4-methyl-zymosterol (Zhou et al., 2023). High expression of NSDHL protein has been found in tissues that synthesize steroids (Caldas and Herman, 2003), while deficiency of NSDHL dramatically impairs cholesterol biosynthesis in various species (Liu et al., 1999; Liu et al., 1999). Herein, we demonstrate that NSDHL was upregulated after the treatment of the CL with LPS during the mid-luteal phase of the estrous cycle. Considering that cholesterol serves as a precursor molecule for the synthesis of progesterone, we can assume that the upregulation of NSDHL might potentially impairs the production of this steroid by the luteal cells. The increased expression of a protein related to cholesterol synthesis in response to LPS could be explained by the involvement of cholesterol in the synthesis of inflammatory mediators–prostaglandins and leukotrienes (Blankenship et al., 1986). Cholesterol has also been demonstrated to play an important role in enhancing Toll-like receptor (TLR) signaling and the activation of NLRP3 inflammasome–crucial defense mechanisms triggered by LPS (Tall and Yvan-Charvet, 2015). Furthermore, our ELISA results showed that LPS treatment also increased progesterone levels in the CL tissue. This finding suggests a complex interaction between inflammatory signaling and steroidogenesis. The increase in progesterone levels may indicate a compensatory mechanism in response to inflammation. Progesterone is known to have anti-inflammatory properties, and its increased synthesis could be a physiological attempt to mitigate the inflammatory response induced by LPS (Fedotcheva et al., 2022). This emphasizes the potential role of NSDHL in mediating both inflammatory and steroidogenic pathways within the CL.

Numerous studies suggest that reactive oxygen species (ROS) are key factors in determining the CL lifespan and that antioxidants play an important role in the CL physiology by controlling the proper timing of the phases of the estrous cycle (Behrman, 2001; Sugino, 2005; Al-Gubory et al., 2008). Apoptosis induced by ROS is involved in the mechanisms of CL regression that occurs in the luteal phase of the menstrual/estrous cycle (Al-Gubory et al., 2012). The balance between ROS and antioxidants is particularly important in the CL, as its high metabolic rate leads to a high consumption of oxygen and energy substrates resulting in high production of ROS and greater exposure of luteal cells to high ROS concentrations (Al-Gubory et al., 2012). Furthermore, LPS is known to increase ROS production in various type of cells (Pal et al., 2022). Our results present that LPS upregulated the expression of one of the most important antioxidants–glutathione S-transferase alpha 1 (GSTA1) – in the CL during the mid-luteal phase of the estrous cycle in pigs. Increased expression of this enzyme under the influence of LPS was frequently detected in various cells (Xu et al., 2017). Our results provide insights into the detoxification processes in response to LPS in the CL. The upregulation of GSTA1 observed in this study highlights a crucial adaptive mechanism to mitigate the harmful effects of increased ROS production. GSTA1 is known to play a central role in cellular defence by conjugating reduced glutathione with reactive intermediates, facilitating their neutralization and elimination (Wróblewska et al., 2023). This process not only prevents cell damage but also supports the maintenance of metabolic homeostasis in the luteal cells.

Macrophages and neutrophiles play a central role in the immune response to LPS as they are essential for phagocytosis and elimination of bacteria (Su et al., 2020). Therefore, its precise regulation ensure effective elimination of microorganisms, limit injury and prevent severe side effects (Muraille et al., 2014). It is known that the inflammatory phenotype of macrophages is significantly influenced by glucose metabolism (Orozco et al., 2012). UDP-glucose has been reported to be a very stable proinflammatory mediator whose levels are strongly related to immune cell recruitment and glycogen metabolism, which play an important role in immune regulation and inflammatory response (Zhang et al., 2024). Recent studies have shown that glycogen as well as the expression of enzymes involved in glycogen biosynthesis, including UDP-glucose pyrophosphorylase 2 (UGP2), are significantly increased in proinflammatory macrophages (Ma et al., 2020). Neutrophils have also been reported to utilize UGP2 to increase their glycogen stores during LPS-induced inflammation, thereby enhancing their energy reserves (Sadiku et al., 2021). In addition, UDP-glucose activates the P2Y14 receptor, whose signalling directs macrophages toward an inflammatory phenotype (Ma et al., 2020) and promote neutrophiles recruitment to the tissue (Sesma et al., 2016). Our findings show that LPS treatment of the CL during the mid-luteal phase of the estrous cycle upregulates the expression of UGP2 – an enzyme that catalyses the synthesis of UDP-glucose from glucose-1-phosphate. To our knowledge, this is the first time we have demonstrated the potential role of UGP2 in mediating inflammation in the CL. This study highlights UGP2 as a promising target for further investigation of its role in immune regulation in the CL. Interestingly, the present study has shown that LPS decreases glucose levels in the CL tissue. Research shows that stimulation with LPS increases the metabolic activity of macrophages and neutrophiles (Schuster et al., 2007; Orozco et al., 2012). In addition to immune cells, glucose is used by other cell types, such as endothelial cells, which are also involved in inflammatory processes. Endothelial cells are essential for the recruitment of immune cells to the site of inflammation and for maintaining the integrity of the vascular barrier, processes that are highly energy-dependent and influenced by the availability of glucose (Danese et al., 2007; Gero, 2018). These cells may consume more glucose to support the energy demands of the inflammatory process, leading to metabolic reprogramming that depletes glucose reserves. Simultaneously, in response to the increased glucose demand in the CL tissue, there is an increased expression of one of the key enzymes responsible for glucose synthesis–UGP2.

The study also has certain limitations. While the enrichment analysis based on the 12 DRPs provides valuable insights, we acknowledge that the relatively small number of input proteins limits the scope and statistical power of the GO analysis. This limitation, driven by the size of the dataset, may affect the strength of the functional enrichment results. Consequently, the interpretations of the identified biological processes and pathways should be approached with caution, as they may not fully capture the broader physiological context. Another limitation of the study is the use of a single dose of LPS. This dose was selected based on previous studies that identified it as reliably inducing an immune response without causing excessive toxicity or mortality (Magata et al., 2014; Shimizu et al., 2016; Gram and Kowalewski, 2022), as well as our own research, where it effectively induced changes in the expression of pro-inflammatory genes such as *TNFSF14*, *NLRP6*, *IL-6*, and *BMX* in the CL (Mierzejewski et al., 2023). However, in the present study, this dose of LPS did not significantly affect proteins directly involved in the immune response. Differences between transcriptomic and proteomic findings are often attributed to post-transcriptional regulation or differential stability of mRNA and proteins. Nevertheless, these results suggest that future studies should focus on various doses effects to gain a deeper understanding of LPS-induced changes in the porcine CL.

In conclusion, this study provides the first comprehensive data on the regulation of proteins in the corpus luteum under *in vitro* stimulation with LPS. The results demonstrate that LPS affects progesterone steroidogenesis, decreases glucose levels and alters its metabolism, as well as influences the tricarboxylic acid (TCA) cycle and detoxification processes in the porcine CL. Furthermore, the study highlights the novel possible roles of UGP2, SUCLG2, and NSDHL in response to LPS in the CL. These findings illustrate the multifaceted effects of LPS on the CL physiology and underscore its potential impact on the ability of the CL to support successful reproduction.

## Data Availability

The data presented in the study are deposited in the MassIVE repository, accession number MSV000094426.

## Supplementary material

SUPPLEMENTARY TABLE S1 All identified proteins in porcine corpus luteum during LPS-induced inflammation.

SUPPLEMENTARY TABLE S2 Differentially regulated proteins in porcine corpus luteum during LPS-induced inflammation.

SUPPLEMENTARY TABLE S3 Gene Ontology analysis of differentially regulated proteins in porcine corpus luteum during LPS-induced inflammation.

SUPPLEMENTARY TABLE S4 KEGG analysis of differentially regulated proteins in porcine corpus luteum during LPS-induced inflammation.
